# Chronic Implant-Related Bone Infections—Can Immune Modulation be a Therapeutic Strategy?

**DOI:** 10.3389/fimmu.2019.01724

**Published:** 2019-07-23

**Authors:** Elisabeth Seebach, Katharina F. Kubatzky

**Affiliations:** Department of Infectious Diseases, Medical Microbiology and Hygiene, Heidelberg University Hospital, Heidelberg, Germany

**Keywords:** chronic implant-related bone infection, osteomyelitis, bacterial infection, biofilm, immune modulation, MDSCs, T cells, immune checkpoint molecules

## Abstract

Chronic implant-related bone infections are a major problem in orthopedic and trauma-related surgery with severe consequences for the affected patients. As antibiotic resistance increases in general and because most antibiotics have poor effectiveness against biofilm-embedded bacteria in particular, there is a need for alternative and innovative treatment approaches. Recently, the immune system has moved into focus as the key player in infection defense and bone homeostasis, and the targeted modulation of the host response is becoming an emerging field of interest. The aim of this review was to summarize the current knowledge of impaired endogenous defense mechanisms that are unable to prevent chronicity of bone infections associated with a prosthetic or osteosynthetic device. The presence of foreign material adversely affects the immune system by generating a local immune-compromised environment where spontaneous clearance of planktonic bacteria does not take place. Furthermore, the surface structure of the implant facilitates the transition of bacteria from the planktonic to the biofilm stage. Biofilm formation on the implant surface is closely linked to the development of a chronic infection, and a misled adaption of the immune system makes it impossible to effectively eliminate biofilm infections. The interaction between the immune system and bone cells, especially osteoclasts, is extensively studied in the field of osteoimmunology and this crosstalk further aggravates the course of bone infection by shifting bone homeostasis in favor of bone resorption. T cells play a major role in various chronic diseases and in this review a special focus was therefore set on what is known about an ineffective T cell response. Myeloid-derived suppressor cells (MDSCs), anti-inflammatory macrophages, regulatory T cells (T_reg_s) as well as osteoclasts all suppress immune defense mechanisms and negatively regulate T cell-mediated immunity. Thus, these cells are considered to be potential targets for immune therapy. The success of immune checkpoint inhibition in cancer treatment encourages the transfer of such immunological approaches into treatment strategies of other chronic diseases. Here, we discuss whether immune modulation can be a therapeutic tool for the treatment of chronic implant-related bone infections.

## Introduction

Primary hip and knee arthroplasties belong to the most successful surgeries of this century (>1,000,000/year in the U.S.) and the numbers of surgeries are rising due to demographical changes (1). Concomitantly, the number of revision surgeries and associated complications is increasing. Prosthetic joint infections (PJIs) are one of the most feared complications that often result in revision of the artificial joint with serious consequences for the patients and high costs for the respective health systems (1, 2). For primary arthroplasty the incidence of infection ranges between 1 and 2% depending on the register (1, 3). A current study states the risk of re-infection after PJI-induced revision surgery at around 8% for hips (4) and knees (5), but also much higher values (up to 57.1%) are published (6, 7). In trauma-related bone reconstructions (2,000,000/year in the U.S.), fracture-related infections (FRIs) associated with osteosynthetic stabilization are a major problem as the surgery field is often contaminated due to bacterial access through open wounds and broken bone that penetrates the skin (open fractures). This leads to an infection risk ranging from 10% (8, 9) to 50% depending on the fracture type (10). Thus, chronic implant-related bone infections are a serious burden in current and future health care.

### Homeostasis of Bone

Bone is a dynamic organ undergoing constant remodeling in order to maintain homeostasis of bone formation and degradation, and to preserve bone mass. Bone remodeling is organized by the interplay between bone forming osteoblasts (OBs) and bone resorbing osteoclasts (OCs). OBs differentiate from mesenchymal stromal cells (MSCs) that reside within the bone marrow, whereas OCs develop from myeloid precursor cells. Osteoclastogenesis is regulated through the osteoprotegerin (OPG)/receptor activator of NF-κB (RANK)/RANK-Ligand (RANKL) pathway. OPG serves as a negative regulator of osteoclastogenesis that inhibits the RANK—RANKL interaction via binding of RANKL [reviewed in (11, 12)]. Bone homeostasis depends on the local cytokine milieu. While inflammation is necessary to induce physiological bone healing (13), it can lead to increased bone resorption under pathological situations such as bone infections (14).

### Definition of Bone Infections

Osteomyelitis is an infection of the bone that is characterized by an inflammatory reaction and destruction of bone due to bacterial colonization of the bone itself, the bone marrow and the surrounding tissue. Osteomyelitis can occur by local spread of bacteria from an adjacent, contaminating source caused by trauma or bone surgery; or secondary to a vascular undersupply as it is mostly the case in diabetic foot ulcers. Hematogenous osteomyelitis is caused by bacteria, which come from a source of infection localized somewhere else in the body (e.g., a dental infection) and enter the bone via the blood stream (15, 16). PJIs caused by hematogenous seeding of the prosthesis often appear a long time after bone surgery (late bone infection: >2 years after surgery), whereas contamination during implantation of the medical device or during hospitalization before the wound has closed usually leads to early (<3 months after surgery) or delayed post-operative infections (3 months−2 years after surgery) (2). Zimmerli and Sendi further suggest a clinically more relevant classification that is used as a guide for surgical management. Here, PJIs are defined as early post-operative when symptoms occur within 1 month and are called chronic when diagnosed later than 1 month after surgery. Hematogenous PJIs are classified as acute when symptoms occur <3 weeks after a former uneventful post-operative period and chronic when symptoms persist for over 3 weeks (17). The predominantly isolated bacteria are part of the physiological skin microflora, such as *Staphylococcus aureus (S. aureus)*, coagulase-negative staphylococci and enterococci (1, 18). Early and acute symptoms of infection, such as pain, warming and swelling of the site of infection and fever, are mostly associated with highly virulent bacteria like *S. aureus*; whereas less virulent bacteria, such as *Staphylococcus epidermidis (S. epidermidis)*, cause more subtle symptoms typical for a low-grade inflammation that often are not diagnosed before infection chronicity (2). FRIs are mostly caused by inoculation of bacteria through an open wound/penetrated skin or through the surgical access needed for osteosynthetic bone reconstruction with *S. aureus* being the primary causative agent (19). At present, they are defined as early when occurring <2 weeks, delayed at 3–10 weeks and late >10 weeks after implantation of the osteosynthetic device (17, 20). However, the criteria for FRIs that can be used as guidelines for clinical management as they are established for PJIs are still under discussion (21). The early and acute states of osteomyelitis are characterized by bacterial colonization of the bone, pus formation, vascular undersupply and a strong inflammatory immune response associated with fever, pain and swelling (15, 16). The resulting increased levels of pro-inflammatory cytokines, such as tumor necrosis factor alpha (TNF-α), interleukin-1 beta (IL-1β) and IL-6, induce tissue destruction and a shift toward osteoclastogenesis and bone resorption (14). At this stage, a prompt and aggressive antibiotic and surgical treatment is generally sufficient to clear the infection. Unsuccessful treatment however results in the manifestation of a chronic bone infection, which is characterized by persistence of bacteria, areas of dead bone, so-called sequestra, periosteal new bone formation, fistula and low-grade inflammation. The recurrence of infection with fever is a clear sign for a chronic progression of the disease (15, 16) and depends on different bacteria reservoirs. *S. aureus* is known to survive intracellularly within non-professional phagocytes such as osteoblasts (22), an immune evasion mechanism still controversially discussed for *S. epidermidis* (23–25). A current study showed that *S. aureus* colonizes the canaliculi and osteocyte lacunae of living cortical bone (26). Furthermore, many bacteria are able to form sessile communities; referred to as biofilms, which preferentially colonize dead bone and foreign devices (17, 27). Biofilms evade bacterial clearance through the immune system and antibiotic treatment and therefore are one key characteristic of chronic implant-related bone infections and a major cause for bacterial persistence (28, 29). Current treatment strategies aim to eradicate biofilms to reduce the risk of re-infection.

### Current Treatment Concepts

Current treatment concepts are based on the surgical removal of the infected tissue and strict antibiotic treatment to reduce bacterial burden as much as possible (17). Antibiotic regimens depend on the result of susceptibility testing of isolated cultures and should be administered for a total duration of 6–12 weeks. In the case of Staphylococcus subspecies, treatment guidelines recommend the use of rifampin, which is effective against biofilm-embedded bacteria, in combination with an intravenously administrable antibiotic for 2 weeks followed by an oral antibiotic therapy. For Methicillin-resistant strains, the combination of rifampin with vancomycin is recommended (20, 30). Surgical treatment of PJIs includes debridement with implant retention and one- or two-stage exchanges with placement of an antibiotic-laden spacer between the explantation and re-implantation of the prosthesis for up to 8 weeks. The procedure applied mainly relies on the time-point, when an implant-related bone infection is diagnosed. In early/acute infections the biofilm is still immature and the infection can be eradicated with retention of the implant. The success rate of this procedure is >80% when the implant is stable and the causative pathogen is susceptible to antibiotic treatment. Otherwise, in the case of Methicillin-resistant *S. aureus* (MRSA) or after chronic manifestation of infection associated with mature biofilm, for example, the foreign device has to be exchanged (30, 31). In FRIs, the decision for retention, exchange or removal of the implant mainly depends on the onset of infection (early-delayed-late), the type of fixation device and fracture consolidation. Here, infection clearance can be achieved because the foreign material can be removed after bone bridging has occurred. Until then, the stability of the bone fracture needs to be preserved meaning that after extensive debridement, external fixation and bone reconstruction may be required. Local delivery of antibiotics either by non-resorbable bone cement or degradable bone graft materials can be beneficial (19, 20). All of these approaches are associated with tremendous consequences for the patients with long hospital stays, repeated surgeries and an impairment of limb function between explantation and re-implantation of the devices. Due to the enhanced tolerance of biofilm-embedded bacteria against most antibiotics and the existence of dormant cells within biofilms, bone niches and/or host cells, treatment approaches often do not end in complete clearance of the pathogen and re-infection occurs frequently (32, 33). As a last consequence, this can lead to non-healing bone defects (non-union), stiffening of the affected joint or even amputation of the infected limb (20, 34).

### Role of the Implant and Biofilm Formation

The implant itself represents a major risk factor for the initial development and chronic progression of osteomyelitis and recurrence of infection. In a tissue cage model in guinea pigs, the presence of a foreign material decreased the required infection dose from >10^8^ CFUs *S. aureus* to 10^2^ CFUs (35). Also in rats, infection doses as low as 10^2^ CFUs *S. aureus* were sufficient to induce implant-associated bone infections without any further promoter such as soft tissue trauma or bone injury (36). One reason for this increased susceptibility is that the implant adversely affects the immune system by activating neutrophils, phagocytic cells, and the complement system. This results in an inflammatory and cytotoxic local environment that causes cell death and tissue damage (37). In this immune-compromised environment, successful clearance of bacteria by the host defense does not take place. Bacteria additionally profit from the foreign material as their surface structures serve as an attractive source for bacterial attachment that facilitate the transition from the planktonic to the biofilm stage (38). The concept of “race to the surface” describes the balance between tissue integration and bacterial colonization of an implant. The success of its implantation depends on the immediate interaction with host cells and the integration within the respective tissue (osseointegration in case of orthopedic devices), which prevents bacterial adhesion and biofilm formation. If bacterial colonization occurs first, tissue integration is impaired and bacteria can persist by forming biofilms (39, 40).

**Figure 1:**
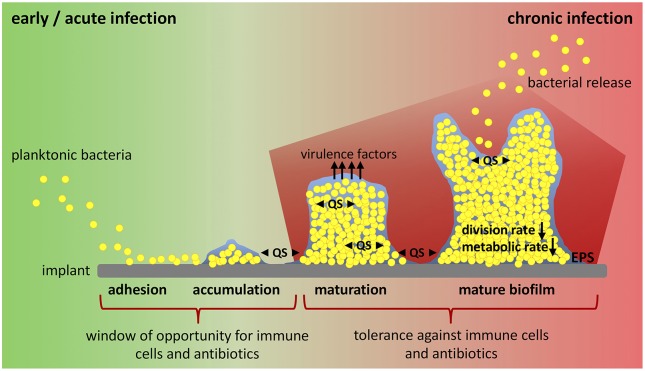
Biofilm formation and window of opportunity for an effective clearance of bacteria. Implant-related bone infections are defined as early and chronic post-operative or acute and chronic hematogenous depending on the time interval between implantation of the medical device and onset of symptoms. Early and acute infections are associated with immature biofilms, whereas mature biofilms play a role in chronic situations. Biofilm formation starts when planktonic bacteria adhere to the implant surface. Attached bacteria then accumulate and start to produce biofilm. During biofilm maturation bacteria strongly multiply, build up further biofilm, and release virulence factors. Mature biofilm shows a high bacterial density with low division rate and decreased metabolic activity (persister cells). Release of planktonic bacteria by biofilm disassembly can lead to recurrence of infection. All these steps are mediated by an intercellular signaling system referred to as quorum sensing (QS). There is only a small window of opportunity for immune cells and antibiotic treatment to successfully clear bacteria and prevent biofilm formation and infection persistence. Biofilm maturation however is characterized by increasing tolerance against immune cells and antibiotics and leads to chronicity of infection. EPS, extracellular polymeric substance; QS, quorum sensing.

In the presence of a medical device, biofilm formation starts with the adhesion of planktonic bacteria to the implant surface, a process mediated by hydrophobic, electrostatic and van der Waals interactions that allows unspecific attachment (Figure 1). Directly after insertion into the body, the implant is coated with serum and tissue proteins. This allows specific attachment of bacteria by bacterial adhesion molecules (microbial surface components recognizing adhesive matrix molecules, MSCRAMMs) that bind to host proteins such as collagen and fibronectin (41). After the initial colonization, bacteria begin to produce a biofilm consisting of exopolysaccharides, proteins, lipids and nucleic acids that form a protective, slimy layer around them (extracellular polymeric substance, EPS). This effectively shields the included bacteria from immune cells and antibiotics (28, 38). Polysaccharide intercellular adhesin (PIA), for example, is a glycosaminoglycan of the EPS that mediates cell-cell adhesion and aggregation of bacteria in staphylococci biofilms (42). Immature biofilms are found in early post-operative and acute hematogenous infections (30). Biofilm maturation is characterized by biofilm growth, bacterial multiplication and production of additional virulence factors (28). Mature biofilms have a high bacterial density and are a constant source of bacterial spreading (43). They are associated with chronic infections (30). Biofilm formation and maturation, production of virulence factors and release of bacteria by mature biofilm are mediated by quorum sensing (QS) signaling systems (28, 44, 45). QS allows cell-to-cell communication between bacteria due to the release of small molecules called “autoinducers”. By this, bacteria are able to determine their population density and react on environmental changes in a population-wide manner (46, 47). Within the hostile environment of mature biofilms, bacteria differentiate into a non-growing phenotype called “persister cells”. This dormant cell population is highly tolerant to antibiotics and contributes to the chronicity and the risk of re-infection of implants (41, 48). Another bacteria phenotype associated with recurrent bone infections are small colony variants (SCVs). SCVs are a metabolically inactive and slow-growing form of bacteria that forms due to defects in electron transport and thymidine biosynthesis (49). Mainly, SCVs are related to intracellular persistence of bacteria as they survive within host cells, but their contribution to biofilm formation and antibiotic tolerance is also discussed (50, 51). In addition to its function as a physical barrier and environment for SCV and persister cell formation, it is hypothesized that biofilm and embedded bacteria affect the local immunological environment in favor of decreased bacterial killing and enhanced persistence (37, 52, 53). The interaction between the foreign device, bacteria and biofilm dampens the host immune response and is one major reason for the ineffective elimination and chronicity of implant-related bone infections (37). Thus, the investigation of endogenous defense mechanisms has moved into focus and the possibility to modulate a misdirected host immune response might provide an attractive target for innovative therapeutic strategies against chronic implant-related bone infections.

The aim of this review is 2-fold: First to summarize the immune response against implant-related bone infections highlighting the transition from acute to chronic infection defined by the presence of biofilm. Secondly, to examine immune modulatory interventions that have been applied for the treatment of other chronic diseases and discuss their feasibility and application for treating chronic implant-related osteomyelitis.

## Immune Response Against Chronic Implant-Related Bone Infections

In the presence of planktonic bacteria, polymorphonuclear neutrophils (PMNs) and macrophages (Mϕs) infiltrate the site of infection. Here, they are activated via binding of pathogen-associated molecular patterns (PAMPs) to the respective pattern recognition receptors (PRRs) such as toll-like receptors (TLRs), which results in the activation of transcription factors such as the nuclear factor “kappa-light-chain-enhancer” of activated B-cells (NF-κB) [reviewed in (54, 55)]. As a consequence, the cells generate an inflammatory environment by secretion of pro-inflammatory cytokines and contribute to bacterial killing by release of antimicrobial peptides, generation of reactive oxygen (ROS) and nitrogen species (NOS) and phagocytosis. Furthermore, PMNs form extracellular fibril matrices consisting of granule proteins and DNA that helps to trap bacteria for further degradation (neutrophil extracellular traps, NETs) [reviewed in (52)]. Thus, in the absence of foreign materials, the innate immune system is usually able to control infection at the planktonic stage leading to bacterial clearance and effective prevention of infection progression.

In the case of implant-related infections, the implant is recognized as a foreign body that induces an innate immune reaction. The release of anti-microbial peptides, ROS, NOS, and NETs and “frustrated” phagocytosis of the non-phagocytosable material leads to cell exhaustion, cell death, and tissue damage. Thus, an immune compromised environment with reduced bacterial killing is established around the implant [reviewed in (37, 41, 56)]. Additionally, the implant creates a niche for bacteria to evade the host defense by hiding in structural pores of the surface that are inaccessible for the larger immune cells (41). So, the foreign material makes clearance of planktonic bacteria ineffective, which ultimately results in bacterial persistence and chronicity of infection.

**Figure 2:**
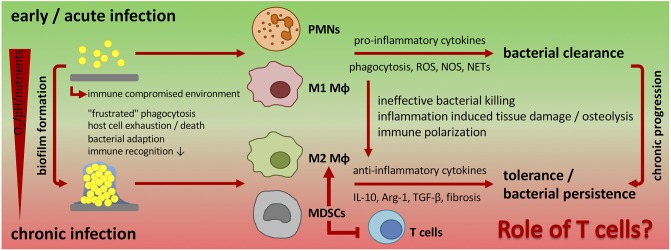
Changing immune response during biofilm formation and chronic progression of implant-related bone infections. Planktonic infections are usually spontaneously cleared by the innate immune system. Neutrophils and classically activated (M1) macrophages are the pre-dominant cell populations that induce a pro-inflammatory cytokine milieu, release antimicrobial products, and phagocytose bacteria. In implant-associated infections the foreign material itself induces an immune reaction. As a result, an immune compromised environment around the implanted device is established that is characterized by an ineffective immune response against the non-phagocytosable material, dysfunction of immune cells and immune cell death. Bacteria take advantage of the foreign material and the impaired immune reaction and start to colonize the implant and form a biofilm. Biofilm-embedded bacteria can adapt to the host defense mechanisms, which results in a decreased immune recognition and enhanced bacterial survival and persistence. The unresolved inflammation is then associated with tissue damage and in the case of bone infections with osteolysis. Additionally, biofilm formation influences the local environment and induces a hypoxic, nutrient-deprived and acidic milieu that further impairs immune cell function. As a consequence, biofilms skew the immune system toward an anti-inflammatory response with a pre-dominantly alternative (M2) macrophage polarization and a high number of immune suppressive MDSCs that are known to inhibit T cell immunity and to induce immune tolerance. Ultimately, this leads to chronicity of infection. The role of T cells in the defense against chronic implant-associated infections is not fully understood and only a few studies focus on this topic. PMNs, polymorphonuclear neutrophils; Mϕ, macrophage; MDSCs, myeloid-derived suppressor cells; ROS, reactive oxygen species; NOS, nitrogen species; NETs, neutrophil extracellular traps; IL-10, interleukin-10; Arg-1, arginase-1; TGF-β, transforming growth factor-beta.

In a mouse model of chronic implant-associated *S. aureus* osteomyelitis, it was shown that biofilm formation on contaminated implants already started on the first day after surgery. Between days 3 and 7, a strong proliferation of bacteria and biofilm growth took place, and the maturation of biofilm reached its maximum around day 14, when proliferation declined, and bacterial dispersal became apparent. The biofilm then stayed stable over the remaining study period for up to 56 days (57). This means that the planktonic window in which an effective bacterial clearance could take place is rather small and that during the course of implant-related bone infections, the immune system is almost entirely confronted with biofilm (Figure 1). The following findings explain at least in part the immune privileged nature of mature biofilms (Figure 2).

The biofilm itself plays an important role in shielding the embedded bacteria against the immune cells and protects the bacteria from immune cell recognition. Mature biofilm consists of a dense extracellular polymeric matrix, which is difficult to penetrate and engulf by phagocytes (58). On the other hand, the EPS contains PAMPs, which normally induce a pro-inflammatory immune response through TLR signaling. However, in the context of biofilms, exopolysaccharides such as PIA, which represent the main matrix component, are associated with immune evasion and protection against innate defense mechanisms (52, 59). Extracellular DNA (eDNA) is an EPS component that consists of eukaryotic DNA from host cells (e.g., through NET formation by PMNs) as well as prokaryotic DNA released by QS-controlled autolysis of bacteria. eDNA has an important role in stabilizing the biofilm matrix and in horizontal gene transfer (52, 60). Bacterial DNA is highly immunogenic and can be recognized by TLR9 (61). For *S. aureus* biofilms however, it was shown that biofilms evade TLR2 and TLR9 recognition. Possible explanations are that the exposure of PAMPs due to the biofilm-shielded bacteria is reduced, and polysaccharides of the biofilm EPS may interfere with TLR-ligand engagement (58, 62). In *Pseudomonas aeruginosa* biofilms, eDNA seems to induce a pro-inflammatory and anti-microbial neutrophil response, as neutrophil activity against *in vitro* biofilms is reduced after DNase treatment (63). However, eDNA also induces increased tolerance against anti-microbial peptides (64). Besides physical and chemical protection, biofilm formation leads to an acidic, hypoxic and nutrient-deprived local environment, which alters immune cell metabolism and activation (65). The release of toxins by the biofilm embedded bacteria further impairs immune cell function and induces cell death (66, 67).

Biofilm is not completely protected against recognition by phagocytic cells (68). *In vitro* data indicate that leukocytes are able to adhere to biofilms and penetrate them under laminar-shear conditions. This is followed by the production of pro-inflammatory cytokines in response to young and mature biofilms; however, the cells were not able to phagocytose the biofilm-embedded bacteria (69). In samples of patients with implant-associated bone infections, Wagner et al. isolated highly activated PMNs, which showed a reduced ability to migrate and a high production of superoxides. The authors concluded that like in planktonic infections, PMNs infiltrate the site of infection and get locally activated but then are unable to effectively clear the biofilm embedded bacteria. Instead, PMNs remain at the site of infection where they release cytotoxic products that contribute to host tissue destruction but do not effectively control the infection (70, 71). Other *in vitro* experiments confirmed the release of granule proteins and DNA by PMNs as a response to biofilm exposure, but in contrast to the study of Leid et al., they could also observe phagocytosis of biofilm bacteria (72). Effective phagocytosis normally depends on the opsonization of bacteria by antibodies and complement factors (73). In contrast to planktonic bacteria, phagocytosis of biofilm by PMNs seems to be independent of opsonization as serum treatment of biofilms did not enhance bacterial uptake. However, reduced deposition of IgG and C3b on biofilm-embedded bacteria contributes to their ineffective killing by PMNs, which may be due to other mechanisms such as a decreased ROS production (74, 75). The biofilm destruction by PMNs was dependent on its maturation stage: whereas immature biofilm (day 2 and 6) was infiltrated and cleared by PMNs at least *in vitro*, mature biofilm (day 15) was shown to be more tolerant against the host immune response (76). This can be explained by the increased biofilm mass making it more difficult for the immune cells to penetrate and engulf the biofilm, but also by an altered gene expression profile of the biofilm embedded bacteria as a reaction to attacking phagocytic cells. Up-regulation for example of the accessory gene regulator (*agr*) locus, which encodes for a staphylococci QS system that activates multiple pathogenicity factors, leads to increased tolerance against immune cell killing and phagocytosis (77, 78). Data from a mouse post-arthroplasty infection model revealed that the recruitment of neutrophils to the site of infection depends on IL-1β. Moreover, the respective knock-out mice showed decreased numbers of neutrophils with more biofilm formation indicating that neutrophils reduce biofilm burden at least to some extent (62). Consistent with these findings, IL-1β expression was decreased during biofilm infection in a mouse catheter-biofilm model (58). Macrophages can either be activated via the classical route which results in a more pro-inflammatory subtype (M1) related to bacterial killing, or via the alternative route which induces a more anti-inflammatory/regulatory and pro-fibrotic subtype (M2) (79). In the mouse catheter-biofilm model, it was shown that biofilm skews infiltrating macrophages from the M1 toward the M2 subtype, as evidenced by a decrease in inducible nitric oxide synthases (iNOS) and an increase in arginase-1 (Arg-1) production. Ultimately, this induced an anti-inflammatory and more pro-fibrotic response preventing effective phagocytosis and bacterial killing (58). The deposition of a fibrotic matrix around the biofilm associated with an alternative macrophage response prevented immune cells from infiltrating the site of infection, which further promoted bacterial persistence. This biofilm-mediated immune suppression was overcome by an early administration of classically activated (M1) macrophages or the treatment with the C5a receptor agonist EP67, which induces a pro-inflammatory macrophage phenotype and indeed resulted in reduced biofilm formation (80). The mechanistic details of how biofilms can polarize macrophages are not completely understood, but one explanation can be an altered immunometabolism. Planktonic bacteria pre-dominantly induce aerobic glycolysis, which provides necessary intermediates for anabolic processing of pro-inflammatory effector molecules such as ROS and NO. Biofilms instead lead to a more anti-inflammatory response, which is generally associated with oxidative phosphorylation (OxPhos). Biofilm formation changes the environmental conditions, which alters the metabolic profiles of macrophages toward OxPhos and anti-inflammation [reviewed in (65)].

Myeloid-derived suppressor cells (MDSCs) are described as a heterogeneous cell population consisting of immature monocytes (M-MDSCs) and granulocytes (G-MDSCs) initially found to suppress T cell activation (81). Typically, these cells differentiate into neutrophils, Mϕs and dendritic cells (DCs) at the site of inflammation, but under chronic conditions such as cancer or chronic infections, respectively, MDSCs arrest in an immature state and promote a negative regulation of the immune system (82). By this, MDSCs have an important role in keeping the balance between long-lasting inflammation and tissue damage, but also contribute to disease chronicity. The mechanisms behind biofilm-mediated MDSC accumulation and arrest have not been determined yet and are important aspects of future research. The group of Tammy Kielian found a remarkable presence of MDSCs in a mouse orthopedic biofilm model (83) as well as in samples from patients with prosthetic joint infections that underwent revision surgery (84, 85). MDSC levels increased continuously after the onset of biofilm formation and then stabilized after chronic progression of infection (85). MDSCs are known to inhibit the pro-inflammatory activation of macrophages. Antibody-mediated depletion of MDSCs within the mouse model therefore resulted in improved bacterial clearance (83). Enhanced numbers of MDSCs and M2 macrophages were also found in a rat PJI model. Additionally, *in vitro* experiments showed that the biofilm was able to induce the differentiation of M-MDSCs into anti-inflammatory M2-like macrophages (86). Using knock-out models for IL-12 or IL-10, the group of Tammy Kielian showed that the presence of IL-12 was required for the recruitment of MDSCs to the site of infection (85), but that the immune suppressive action of MDSCs was mediated by release of IL-10, a cytokine known to shift macrophage polarization toward an anti-inflammatory phenotype (87). The loss of IL-12 or IL-10 resulted in lower numbers of MDSCs, enhanced presence of pro-inflammatory monocytes, increased bacterial clearance and decreased biofilm burden. Adoptive transfer of wild-type MDSCs restored MDSC influx and immune suppressive action with aggravated disease outcome (85, 87). MDSC-derived Arg-1 only showed a minimal effect on biofilm growth. Instead, Arg-1 seemed to play a role in host immune cell activity against planktonic bacteria, which again confirmed the divergent immune responses against planktonic and biofilm infections (88).

The last step of the biofilm lifecycle is the release of bacteria back into their planktonic stage. By this, the bacteria become re-accessible for antibiotics and host defense mechanisms; however, this can also be linked to the spreading of infection and sepsis (43, 89). Furthermore, there is evidence that bacteria released from mature biofilms induce an increased pro-inflammatory reaction when compared to their planktonic counterparts that further supports inflammation-associated tissue destruction and infection relapse (90).

### Role of T Cells

T cells belong to the adaptive immunity and mediate the specific immune response. They can be divided into cluster of differentiation (CD)4-positive helper T cells and CD8-positive cytotoxic T cells. Cytotoxic CD8 T cells directly eliminate infected cells through the release of cytotoxic proteins. CD4 helper T cells need to get activated by professional antigen presenting cells (APCs) in order to support a cellular and humoral immune response. T cell activation occurs after binding of an antigen- major histocompatibility complex (MHC)-complex to a T cell receptor (TCR) and further requires costimulation by binding of CD28 present on T cells to CD80/86. Depending on the cytokine environment, CD4 helper T cells differentiate into Th1, Th2, and Th17 subtypes as well as regulatory T cells (T_reg_s) (91). The contribution of T cells during the immune response against chronic implant-related bone infections is not fully determined and there are some contradictory data about the presence, effector function and inhibition of T cells at the site of biofilm infections that will be addressed in the following section (Tables 1A,B).

**Table 1A:** T cell response against implant-related bone infections—human studies.

**Research question**	**Approach**	**Major findings**	**References**
Characterization of leukocyte infiltrates and cytokine expression in PJI samples compared to aseptic loosening.	• Samples from endoprosthesis patients with PJI or aseptic loosening were analyzed for leukocyte counts and subtypes (FACS) and cytokines (Multiplex Assay).	➢ Higher leukocyte numbers in infected vs. aseptic samples. ➢ Higher numbers of G-MDSCs in infected vs. aseptic samples (no difference in neutrophils or monocytes). ➢ Reduced T cell numbers in infected vs. aseptic samples (non-significant). ➢ Increased levels of IL-10, IL-6 and CXCL-1 in infected vs. aseptic samples. ➢ **Accumulation of immune suppressive G-MDSCs in PJIs prevents activation of antimicrobial effector mechanisms by this leading to infection persistence**.	(84)
Characterization of leukocyte infiltrates and cytokine expression in PJI and aseptic human samples for comparison with data from a mouse orthopedic infection model.	• Samples from endoprosthesis patients with PJI or aseptic loosening were analyzed for leukocyte counts and subtypes (FACS) and cytokines (qPCR, Multiplex Assay).	➢ Increased MDSC-like and reduced T cell numbers with elevated pro-inflammatory cytokine levels in infected compared to aseptic human samples. ➢ **Comparable immune response during orthopedic biofilm infection between mouse and human system**.	(85)
Analysis of T cell activity in human tissue samples after infectious vs. aseptic implant loosening.	• FACS, histological and gene expression analysis of T cell infiltrates in tissue samples from patients undergoing infectious or aseptic revision surgery.	➢ Increased numbers of CD28^−^CD11b^+^ (activated) CD4 or CD8 T cells in infected samples vs. aseptic samples. ➢ Increased expression of T cell marker CD3 and no differences of monocyte marker CD14 and osteoclast marker cathepsin K in infected vs. aseptic samples. ➢ **Enhanced numbers of activated T cells in implant-associated infection**.	(92)
Characterization of T cell phenotype in chronically infected vs. non-infected bone samples.	• Analysis of cortical bone samples from patients undergoing primary prosthetic surgery (non-infected) and samples from patients undergoing revision surgery (chronically infected) by multiparametric FACS.	➢ Presence of CD4 and CD8 T cells in both samples, increased HLA-DR expression on T cells and reduced T cell proliferation in infected vs. non-infected samples, no T_reg_s or T cell apoptosis in infected samples. ➢ Increase of CD28^−^ CD4 T cells and CD80^+^, CD40^+^ and CD40L^+^ CD4 and CD8 T cells in infected vs. non infected samples. ➢ Increased perforin and CD11b and decreased CD7 expression in CD28^−^ T cells. ➢ Increased number of (long-term activated) cytotoxic CD28^−^ CD4 T cells with reduced proliferation capacity in chronically infected bones.	(93)
Analysis of systemic and local T cell activation in patients with implant-associated bone infections.	• Blood and lavage from site of infection were taken from patients with implant-associated bone infections and analyzed by FACS for T cell activation markers.	➢ Upregulation of CD11b and loss of CD28 on CD4 T cells in blood samples of infected patients compared to healthy donors. ➢ Increased expression of TLR1,2,4 associated with CD11b^+^CD28^−^ CD4 T cells in blood samples of infected patients. ➢ Accumulation of CD11b^+^CD28^−^ CD4 T cells and CD57^+^ CD8 T cells at site of infection. ➢ Increased IFN-γ expression by T cells from site of infection. ➢ **Recruitment and activation of CD4 and CD8 effector T cells in patients with implant-associated bone infections**.	(94)
Analysis of T cell infiltration in patients with implant-associated bone infections compared to patients with sterile joint inflammation.	• Blood and lavage from site of infection were taken from patients with implant-associated bone infections and analyzed by FACS for T cell markers. • As control synovial fluid from patients with rheumatoid arthritis (RA) was used.	➢ Loss of CD62L expression by T cells isolated from the infection or inflammation site compared to respective blood sample. ➢ Shift of local CD4/CD8 ratio toward CD8 in infected and CD4 in RA patients. ➢ Perforin and granzyme B expression by CD8 T cells at site of infection. ➢ Detection of CD28^+^ and CD28^−^ subpopulation in lavage with increased CD11b and CD57 expression on CD28^−^ CD8 T cells. ➢ **Expansion and infiltration of cytotoxic CD8 effector T cells in patients with implant-associated bone infections**.	(95)

*Bold text indicates key finding of the respective study*.

**Table 1B:** T cell response against implant-related bone infections—mouse models.

**Research question**	**Approach**	**Major findings**	**References**
Characterization of invading MDSC subpopulations in a mouse orthopedic biofilm infection model.	• Insertion of a K-wire in femora of C57BL/6 mice and inoculation of 10^3^ CFU *S. aureus* (SA) at the implant tip. • Analysis of infiltrating leukocyte populations by FACS, cytospin, *in vivo* proliferation assay, *in vitro* T cell activation capacity and RNA sequencing on days 3, 7, 14, and 28.	➢ Identification of CD11b^high^ granulocytic MDSCs and CD11b^low^ PMNs. ➢ **G-MDSCs proliferate at the site of biofilm infection and suppress T cell response over the whole course of infection (planktonic and biofilm phase), whereas PMNs show immune suppressive activity only after biofilm development**.	(96)
Monitoring of the immune reaction during sterile or infected bone healing in an implant-stabilized mouse fracture model.	• Fixation of a SA pre-incubated osteosynthetic device (9 × 10^5^ CFU/implant) and creation of an osteotomy in femora of C57BL/6 mice. • Quantitative microbiology and analysis of immune response by histology, FACS, qPCR, and Multiplex Cytokine Assay over 35 days.	➢ Positive cultures over whole period with highest bacterial loads on days 1–3. ➢ Complete bone healing in non-infected controls by day 35, non-union with osteolysis in infected animals. ➢ Minimal inflammatory cell infiltration in controls on day 3 with signs of tissue healing on day 7, increased invasion of inflammatory cells in infected animals on day 3 with strong inflammation/osteolysis on day 7. ➢ Increased cell numbers in lymph nodes and spleen of infected animals. ➢ Increased IL-4 and late IFN-γ expression in controls and increased IL-17, TNF-α, IL-1β, and IL-10 expression in infected animals. ➢ **Bone healing is associated with sustained Th2 and late Th1 and bone infection with a central Th17 and pro-inflammatory response unable to control infection (with decrease in bone healing markers TGF-β and PDGF)**.	(97)
Role of MDSC-derived IL-10 in MDSC-mediated immune suppression in orthopedic biofilm infections.	• Insertion of a K-wire in femora of C57BL/6 wt and IL-10 ko mice and inoculation of 10^3^ CFU SA at the implant tip. • Adoptive transfer experiments of wt MDSCs in IL-10 ko mice. • Analysis of bacterial burden by SA recovery, MDSC and monocyte/Mϕ invasion and cytokine profile by ELISA, FACS, cytokine array and qPCR on days 3, 7, and 14 and analysis of *in vitro*-derived wt and IL-10 ko MDSC activity by T cell proliferation assay.	➢ Infiltrating MDSCs are the main source of increased IL-10 levels in orthopedic implant biofilm infections. ➢ Decreased MDSCs and increased monocyte/Mϕ recruitment in IL-10 ko mice on day 14 with enhanced pro-inflammatory activity of monocytes/Mϕ and decreased bacterial burden. Partly reversible by adoptive transfer of wt MDSCs. No changes in neutrophil and T cell infiltrates in IL-10 ko mice. ➢ Inhibition of T cell proliferation by biofilm-associated MDSCs is independent of IL-10. ➢ **MDSC-derived IL-10 induces an anti-inflammatory monocyte phenotype at the site of biofilm infection that promotes bacterial persistence, but has no direct effect on T cell proliferation**.	(87)
Role of IL-12 in MDSC recruitment and MDSC-mediated immune suppression in orthopedic biofilm infections.	• Insertion of a K-wire in femora of C57BL/6 wt and IL-12 ko mice and inoculation of 10^3^ CFU SA at the implant tip. • Adoptive transfer experiments of wt MDSCs in IL-12 ko mice. • Analysis of bacterial burden by SA recovery, MDSC and monocyte/Mϕ invasion and cytokine profile by ELISA, FACS, cytokine array and qPCR on days 7, 14, 21, and 28, CT and histology and analysis of MDSC activity isolated from site of infection of wt and IL-12 ko mice by T cell proliferation assay. • Comparison of data with human samples of PJIs.	➢ Detection of bacteria during the whole period with strong inflammation of infected tissue and bone destruction. ➢ Increased cytokine (IL-12, IL-1β, TNF-α, and G-CSF) and chemokine levels in infected animals, associated with increased MDSC and neutrophil and reduced monocyte/Mϕ and early T cell invasion compared to aseptic samples. ➢ IL-12 ko mice show decreased MDSC recruitment and decreased cytokine levels with enhanced monocyte and neutrophil infiltration and decreased bacterial burden compared to wt mice. ➢ Adoptive transfer of wt MDSCs in IL-12 ko mice reduces monocyte and neutrophil invasion and leads to increased bacterial burden compared to wt mice. ➢ MDSC isolated from site of infection in IL-12 ko mice are able to inhibit T cell proliferation like MDSC from wt mice. ➢ **IL-12 promotes MDSC accumulation at the site of infection, causing a MDCS-mediated reduction of monocyte and neutrophil invasion. No direct role of IL-12 in activation of immune suppressive MDSC function and T cell proliferation**.	(85)
Role of MDSCs and MDSC-mediated T cell suppression in orthopedic biofilm infections.	• Insertion of a K-wire in femora of C57BL/6 mice and inoculation of 10^3^ CFU SA at the implant tip. • Antibody-mediated depletion of MDSC *in vivo*. • Histological, Multiplex cytokine array, FACS and qPCR analysis of tissue samples, T cell proliferation assay and monocyte co-culture experiments.	➢ Increased numbers of MDSCs in samples of infected animals vs. non-infected controls on day 7. ➢ MDSCs isolated from infected tissue inhibit T cell proliferation and cytokine secretion. ➢ MDSC depletion reduce biofilm burden (mainly by restoring pro-inflammatory activity of monocyte). ➢ **Biofilm-associated MDSCs inhibit T cell response**.	(83)
Prevention of chronicity of an implant-associated biofilm infection through a Th1↓/Th2↑ polarized immune reaction.	• Implantation of SA pre-treated pins (3 × 10^5^ CFU/pin) in tibiae of Th1-biased C57BL/6, Th2-biased Balb/c and STAT6 ko Balb/c mice. • Treg depletion in Balb/c with anti-CD25 and Th1 suppression in C57BL/6 with anti-IL12p40 treatment. • Analysis of bacterial clearance, Treg frequency and local cytokine profile on days 7 and 21.	➢ Spontaneous bacterial clearance in ~75% of Balb/c mice. ➢ Higher levels of IL-4 and IL-10 and T_reg_ frequency in Balb/c and increased neutrophil infiltration in C57BL/6 mice. ➢ STAT6 ko and T_reg_ depletion lead to loss of protection in Balb/c mice. ➢ Anti-IL12p40 treatment induces bacterial clearance in ~40% of C57BL/6 mice. ➢ **Early induction of Th2/T**_**reg**_ **and suppression of Th1/Th17 response protects from chronicity**.	(98)
Investigation of immune response during chronic progression of an implant-associated biofilm infection.	• Implantation of SA pre-treated pins (2 × 10^5^ CFU/pin) in tibiae of C57BL/6 mice. • Analysis of activated immune cells, antibody production and local cytokine up to day 28.	➢ Activation of a CD4 T cell response, early production of Th1-IgG subtype IgG2b and local pro-inflammatory cytokine profile in infected animals. ➢ **An early Th1 and Th17 and reduced T**_**reg**_ **immune response is ineffective to prevent infection and leads to chronicity**.	(99)

*Bold text indicates key finding of the respective study*.

Studies using human tissue samples indicate that CD4 and CD8 T cells are present at the site of implant-related biofilm infections (92, 93). T cells isolated from the infectious samples showed a high proportion of CD28^−^/CD11b^+^ cells that indicates terminally differentiated T effector cells. These cells further produced high levels of perforin and IFN-γ typical for cytotoxic T cells which are classically associated with virus infections (Table 1A) (93–95). Whether this T cell response participates in infection defense or contributes to bone destruction by promoting osteoclastogenesis is yet unknown and has to be addressed in future studies. Mouse models showed that chronic implant-related bone infections can have a pronounced pro-inflammatory Th1 and Th17 response that is unable to clear infections at an early stage (Table 1B) (97, 99). Indeed, an early induction of a Th2/T_reg_ based response was able to prevent chronicity of infection (98). Heim et al. found only low numbers of T cells at the site of orthopedic biofilm infections in human samples (Table 1A) (84, 85) as well as in the corresponding mouse model (Table 1B) (83). The authors explain this with a high presence of MDSCs in their samples (see section above) (83) and they showed that the MDSCs, in particular G-MDSCs, suppressed T cell proliferation throughout the course of infection (83, 96). This fits with the finding of Kumar et al. who reported a reduced T cell proliferation in patient samples from chronic prosthesis infection (93). Along with inhibiting local T cell proliferation, MDSCs were associated with decreased T cell homing to the site of infection by down-regulated L-selectin (CD62L) expression (100), which might additionally explain the low numbers of T cell infiltrates. The mediators behind MDSC-derived T cell suppression are not clear yet, but this seems to be independent of IL-10 and IL-12 (85, 87). Besides that, Heim et al. found that the effects of MDSC-mediated immune suppression were more obvious on phagocytic cells (monocytes and neutrophils) than on T cells. The absence of MDSC action in the orthopedic biofilm mouse model led to an increased influx of monocytes and neutrophils and restored pro-inflammatory activity of these cells and resulted in decreased bacterial burden (83, 85, 87). Interestingly, in the same mouse model, PMNs as well as monocytes also exhibited suppressive activity on T cell proliferation after biofilm had developed (96). Besides playing a major role in innate immunity against pathogens, PMNs are discussed to directly interact with T cells. They are assumed to be able to activate T cells through MHC class II–mediated antigen-presentation as well as to exert an immune suppressive action on T cells by depletion of L-arginine via Arg-1 thereby exhibiting a more MDSC-like phenotype (101). This indicates that biofilm maturation potentially changes the initial pro-inflammatory PMN function toward a more anti-inflammatory action, which might then have an additional impact on the T cell response during infection progression.

Brady et al. compared the immune response in subcutaneous mouse models of acute and chronic implant-related biofilm *S. aureus* infection. By analyzing cytokine and chemokine levels of respective tissues using proteomic arrays, they found increased cytokine levels indicating a promoted pro-inflammatory Th1/Th17 response in their biofilm model. This was associated with down-regulated chemokine levels and decreased T cell homing to the site of infection, creating a strong pro-inflammatory reaction with low T cell infiltration (102). Interestingly, by comparing early (day 7) with late (day 21) biofilm infection in their chronic infection model, they found a similar cytokine response during the course of infection, which did not show any remarkable changes, but simply decreased when the infection become chronic. This is explained partly by the fact that at this time most bacteria are metabolically inactive and production of virulence factors and pro-inflammatory mediators has declined. Further research is needed to investigate possible additional factors that play a role in the dampened response after biofilm formation and chronicity of infection.

The activation of naïve T cells by APCs is an essential step of the T cell response. It is therefore conceivable that an altered APC function can lead to an ineffective T cell immunity against biofilms. Likely, APCs are already impaired by the implant and contribute to the immune compromised environment and increased bacterial colonization. Two biodegradable and biocompatible materials that are known to provoke a normal foreign body response were tested for DC activation and subsequent DC-mediated T cell proliferation and polarization in the presence or absence of *S. aureus* and *S. epidermidis*, respectively (103). The authors found that the biomaterials alone did not induce DC activation and subsequent DC-mediated T cell activation, but in combination with bacteria, DCs had a slightly changed cytokine secretion profile. However, these changes were too small to affect subsequent T cell activation. Thus, the presence of a foreign material does not impair APC-mediated T-cell activation upon bacterial exposure. The altered cytokine secretion by DCs stimulated by bacteria in the presence of a biomaterial could still have an impact on other immune cells like PMNs and macrophages which can promote bacterial survival. In this study only planktonic bacteria were used to stimulate DCs in the presence of a biomaterial. Therefore, the influence of biofilm formed on the biomaterial and potential changes in DC and subsequent T cell activation remain to be investigated.

So far, there are only a few studies that address the T cell response in chronic implant-associated bone infections and results argue for the presence of activated cytotoxic T cells at the site of biofilm formation and an early pro-inflammatory Th1/Th17 response. However, the decreased homing to the site of biofilm infection and a reduced T cell proliferation and potentially impaired function might trigger the formation of biofilm-associated suppressive immune cells.

It has to be taken into consideration that mouse studies that investigate the immune response against implant-related bone infections usually use *S. aureus* to induce biofilm-infections (Table 1B). However, *S. aureus* is a highly virulent pathogen that causes a strong pro-inflammatory Th1 immune response in planktonic infections (102) and early and acute bone infections (30). It needs to be investigated whether the findings also apply for less virulent bacteria like *S. epidermidis* that is associated with less symptomatic but chronic implant-infections. A recent study compared *S. aureus* and *S. epidermidis*-induced implant-associated osteomyelitis in mice (104). This study revealed that *S. aureus* caused osteolysis, reactive bone formation and abscess formation, whereas this was not apparent in *S. epidermidis* infection. Both bacteria colonized the implant and formed biofilm. The findings underline the different roles of *S. aureus* and *S. epidermidis* in chronic implant-related bone infections. In human studies, the cohorts include patients with implant-related bone infections caused by different bacteria and the time point of revision surgery and immune analysis might depend on the virulence of the respective bacteria. So, it is possible that different stages of biofilm infection are within the same cohort. This might explain the apparently conflicting findings in T cell quantities between the different studies (84, 92). Investigations of T cells in implant-related bone infections have been restricted to the evaluation of numbers and types of T cells present at the site of biofilms. The functionality of biofilm-associated T cells and the mechanisms behind the T cell response have not been examined yet. Apparently, there is a need for further research to investigate the insufficient T cell response during biofilm formation and chronic progression of implant-related bone infections in more detail.

### Humoral Immune Response

The identification of a protective humoral immunity (105) and biofilm-associated antigens raised the hope for vaccination strategies (106). Indeed, administration of a multicomponent and protein-based vaccine before bacterial challenge with subsequent antibiotic treatment significantly reduced the risk for infection in a biofilm model of osteomyelitis in rabbits (107). Passive immunization against implant-related osteomyelitis in mice with neutralizing antibodies associated with protective immunity in orthopedic infections led to reduced bacterial burden, osteolysis, and abscess formation, respectively, due to increased opsonophagocytosis of bacterial megaclusters by recruited macrophages (108, 109). A current study showed that a combinatory approach using passive immunization together with antibiotic and surgical treatment was capable of reducing re-infection in a mouse model of MRSA-induced implant-related osteomyelitis, thereby enabling osseointegration and bone healing (110). Despite this promising animal data, unfortunately, attempts to develop an effective vaccination strategy for humans have been unsuccessful so far (111).

### Role of the Bone Environment

Due to the crosstalk between bone and immune cells, cells of the bone environment (OBs, OCs, MSCs) are also involved in the course of bone infection. A pro-inflammatory immune cell environment induces a shift in bone homeostasis toward increased osteoclastogenesis and bone resorption, which is further supported by local osteoblasts that can release pro-inflammatory proteins in response to bacteria (112, 113). Dapunt et al. showed that expression of pro-inflammatory proteins by osteoblasts is not only induced by planktonic bacteria but also by biofilm components. This indicates that OBs not only play a role in the host response against biofilm-associated infections, but also enhance osteolysis associated with these infections (114, 115). Besides an increased osteoclastogenesis, new bone deposition by osteoblasts is reduced as the infectious environment and bacterial internalization lead to decreased mineralization and increased apoptosis of osteoblastic cells (116). Release of internalized bacteria and dying osteoblasts might further impair the immune response against the bacteria.

In the case of osteosynthetically stabilized fractures, implant-associated bone infections impair the healing process and can lead to non-unions. During bone regeneration, the host response against bacteria and biofilm seems to interfere with the naturally occurring immune reaction required to induce the healing cascade. This unresolved pro-inflammatory environment is ineffective to clear the infection and at the same time is detrimental to bone regeneration (97, 117). MSCs as osteogenic precursor cells have an important role in bone healing (118). They are also known to have immune modulatory activity and exert an immune suppressive effect on T cells (119), which might impact the development and progression of bone infections. Indeed, in a rat plate-stabilized ostectomy-model, local implantation of MSCs to improve bone regeneration aggravated implant-associated bone infections (120).

These data indicate that implant-associated bone infections and septic non-unions are characterized by a complex interplay between bacteria, cells of the immune system, and cells of the bone environment.

### Osteoclasts as Immune Competent Cells

Besides being the main players in bone resorption, osteoclasts are part of the immune system and interact with immune cells, especially with T cells [reviewed in (121, 122)]. Interactions are ambilateral with T cells influencing osteoclastogenesis and OCs having an impact on T cell activity. Activated T cells express RANKL which stimulates the differentiation of human monocytes into mature osteoclasts (123). Th17 helper cells and their cytokine IL-17 are shown to enhance osteoclastogenesis, while the Th1 and Th2 cytokines IFN-γ and IL-4 are associated with an anti-osteoclastogenic potential (124, 125). T_reg_s were proven to have an inhibitory effect on osteoclast generation [reviewed in (126)]. In addition to the anti-osteoclastogenic effects of T_reg_-derived cytokines IL-10 and TGF-β, direct cell-cell contact through binding of cytotoxic T-lymphocyte-associated protein-4 (CTLA-4) to CD80/86 on osteoclast precursors inhibits osteoclastogenesis (127) (more information on this are provided in the following section about immune modulation). OCs can function as antigen presenting cells that can activate T cells upon antigen exposure (128). However, a suppressive effect of OCs on the *in vitro* T cell response via the induction of indoleamine 2,3-dioxygenase (IDO) was also described (129, 130). Furthermore, OCs can prime CD8 T cells toward a regulatory phenotype (OC-iTc_reg_) which then again has a suppressive effect on T cell activation and inhibit osteoclastogenesis [reviewed in (131)]. Taken together, it can be said that osteoclast precursors share many of the immune suppressive characteristics that have been associated with MDSCs (121, 132).

So far, research investigating the immunological function of osteoclasts has been done under sterile conditions either in *in vitro* experiments or in animal models of sterile bone loss, such as inflammatory arthritis or osteoporosis. Whether similar findings can be obtained in an infectious setting such as implant-associated bone infections need further investigations.

In summary, the implant as a foreign material as well as the bacteria, especially in form of a biofilm, lead to a dysregulation of the immune response and misbalance of bone homeostasis in favor of bacterial persistence, bone destruction and infection chronicity. We suggest that this impaired osteoimmunological environment represents an attractive target for modulation, making immune therapy an interesting approach for the treatment of chronic implant-related bone infections.

## Modulation of the Immune Response During Chronic Implant-Related Bone Infections

**Figure 3:**
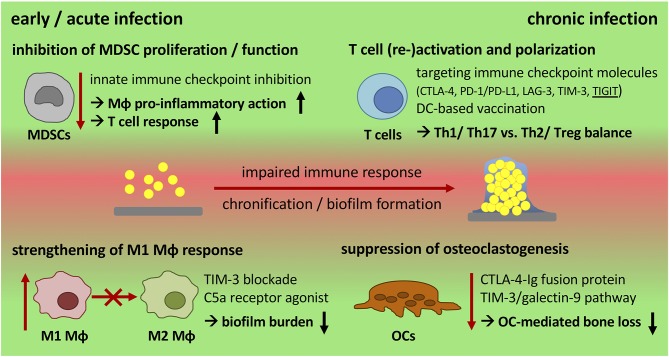
Potential targets for immune modulation during chronic progression of implant-related bone infections. Biofilm formation skews the immune response toward an anti-inflammatory, immune inhibitory and tolerant environment that is associated with high numbers of MDSCs, M2 macrophages, and an ineffective T cell response. Immune modulation by therapeutic intervention offers the possibility to generate a more effective immune response that supports bacterial killing and the reduction of biofilm burden. An early inhibition of MDSC activity and the induction of a more pro-inflammatory (M1) Mϕ response are potential targets to strengthen innate defense mechanisms. Re-stimulation of T cell effector functions by targeting immune checkpoint molecules can overcome T cell dysfunction caused by chronic disease progression and might prevent re-infection after revision surgery. Targeting TIGIT as well as using DC-based vaccination strategies may provide the opportunity to direct T cell polarization toward Th1 or Th2 -dominated responses. When boosting the immune system, its impact on inflammatory tissue destruction has to be considered as a balance between anti-bacterial activity and cytotoxicity is required. CTLA-4 and the TIM-3/galectin-9 pathway are important immune regulators that can also be used as checkpoints to control osteoclast numbers as a means to reduce bone resorption. MDSCs, myeloid-derived suppressor cells; Mϕ, macrophage; DC, dendritic cell; OCs, osteoclasts; ICI, immune checkpoint inhibitor; CTLA-4, cytotoxic T-lymphocyte-associated protein-4; PD-1/PD-L1, programmed cell death protein-1/PD ligand-1; LAG-3, lymphocyte activation gene-3; TIM-3, T cell immunoglobulin and mucin-domain containing protein-3; TIGIT, T cell immunoglobulin and ITIM domain.

Enormous effort has been put into the development of new antibiotics, anti-microbial coatings of the implant, vaccination strategies as well as interruption of the QS system to avoid biofilm formation and chronicity of implant-related bone infections, yet with limited success (32). Modulation of the immune system is a promising field in treating chronic diseases and offers the potential to combine current therapeutic and surgical strategies while strengthening endogenous defense mechanisms. Especially the success of immune therapy in cancer treatment encourages to take a broader view and transfer novel approaches into other diseases. The following section will address what is known about immune therapy in other chronic diseases and discuss whether there are targets for immune modulation that might allow treating chronic implant-related bone infections (Figure 3 and Table 2).

**Table 2:** Potential targets for immune modulation during chronic implant-related bone infections.

		**Target**	**Mechanism**	**Potential benefit for chronic implant-related bone infections**
T cell immunity		CTLA-4	Competitive binding of CD80/86 and inhibition of T cell co-stimulation. Anti-CTLA-4 antibodies restore T cell activation.	Isolated T cells from chronic implant-related bone infections are mostly CD28^−^. Usefulness of CTLA-4 to re-activate T cells after chronicity of infection is therefore questionable.
		PD-1/PD-L1	Induction of effector T cell exhaustion. Blocking this pathway by antibodies restores T cell function.	Role of exhausted T cells in chronicity of bone infections is unclear. Cells of the bone environment (MSCs, OCs) express PD-L1 upon inflammation, therefore inhibition of this pathway might decrease bone cell-mediated T cell suppression.
		LAG-3/TIM-3/TIGIT	Inhibition of APC-mediated T cell activation and Th1/Th17 -mediated T cell response.	Blood T cells from patients with chronic osteomyelitis show increased expression of LAG-3 and impaired proliferation/function. Hence, LAG-3 blockade can increase T cell activation in chronic implant-related bone infections. An early Th2/T_reg_ immunity was shown to prevent biofilm formation and chronicity in a murine orthopedic implant infection model. TIGIT treatment at an early time point can be supportive to clear infections via induction of a Th2-based T cell response.
Innate immunity	MDSCs	Innate IC molecules	Controlling MDSC proliferation and function.	MDSCs are associated with an anti-inflammatory environment in chronic implant-related bone infections. Eliminating MDSCs can prevent unwanted immune suppression and strengthen pro-inflammatory immune reactions.
	Mϕ	TIM-3	Inhibitory receptor on Mϕs, by this suppressing a pro-inflammatory response.	Chronic implant-related bone infections are associated with a shift toward an anti-inflammatory (M2) Mϕ phenotype which supports bacterial persistence. Blockade of TIM-3 can strengthen a pro-inflammatory (M1) Mϕ response and enhance bacterial killing.
		C5a receptor	Binding of C5a receptor influences Mϕ polarization.	Targeting Mϕ polarization via C5a receptor ligands can prevent formation of anti-inflammatory (M2) Mϕs associated with chronic infection. Early treatment with a C5a receptor agonist induces a pro-inflammatory (M1) Mϕ response which leads to reduced biofilm burden in a mouse implant-infection model (80).
	DCs	Antigen presentation	Induction of an antigen-specific T cell immunity and desired T cell differentiation.	The role of DCs in chronic implant-related bone infections is unclear, but DC therapy could allow the generation of biofilm-specific DCs and the induction of a more effective host immune response.
Osteoclastogenesis		CTLA-4	Inhibition of osteoclastogenesis through binding to CD80/86 on monocytes. Administration of a CTLA-4-Ig fusion protein reduces osteoclast numbers.	Bone infections are associated with high numbers of osteoclasts and increased bone resorption, CTLA-4 treatment can reduce inflammation-induced bone destruction.
		TIM-3/galectin-9	Binding of galectin-9 to TIM-3 expressed on osteoclast precursors suppresses osteoclastogenesis.	Targeting the TIM-3/galectin-9 pathway can reduce osteoclast formation and bone loss in chronic implant-related bone infections.

### Immune Regulation During Chronic Diseases

Immune responses are tightly regulated to prevent an unresolved immune reaction, which would lead to long-lasting inflammation and tissue damage. The regulation of this process is mediated by cells of the innate and adaptive immune system including immune suppressive MDSCs, anti-inflammatory (M2) Mϕ and regulatory T_reg_s that help to generate an immune microenvironment characterized by high levels of IL-10, Arg-1 and TGF-β (133). In general, this limits the pro-inflammatory effector phase, which ends with antigen clearance, resolution of inflammation and the induction of an immunological memory. In contrast, disease continuation and long-term exposure to antigens, as it occurs in tumors or chronic infections, induce an enhanced up-regulation of inhibitory molecules by immune cells. Ultimately, this leads to immune cell dysfunction associated with ineffective control and persistence of disease. Upon long-term stimulation, T cells increasingly express inhibitory receptors, known as “immune checkpoint molecules,” of which CTLA-4 and programmed cell death protein-1 (PD-1) are the most prominent members. Binding of their respective ligands expressed on immune and non-immune cells leads to T cells with low or diminished effector functions that are called anergic or exhausted T cells. T cell dysfunction has moved into the focus of interest as it can be reversed by the use of immune checkpoint inhibitors (ICIs), which makes them an attractive target for re-stimulation of the immune response [reviewed in (134)]. Blockade of immune checkpoints has been successfully introduced into certain cancer treatments (135) and is discussed as a treatment option for infectious diseases such as malaria, HIV (136) and sepsis (137). Furthermore, Fc-fusion proteins of immune checkpoint molecules are currently being investigated for their use as immune suppressive therapy e.g., in autoimmune disorders (138). Immune therapy therefore includes immune activating and immune suppressing approaches, both of which represent attractive targets for treatment of chronic infections depending on the local immune environment.

### Immune Activation or Suppression in Chronic Implant-Related Bone Infections

Long-lasting interactions of bacteria, biofilm components, and host cells that occur in chronic implant-related bone infections severely impair the immune response. Thus, immune therapy can be an interesting tool to restore appropriate immune function. As suggested by the literature, chronic implant-related bone infections initially provoke a more pro-inflammatory immunity. This is then dampened to a more anti-inflammatory and immune tolerant response during the chronic course of infection, which prevents tissue damage but also contributes to bacterial persistence. However, pro- and anti-inflammation cannot be simply attributed to different stages of the disease as they occur simultaneously throughout the course of infection. Favoring one above the other would risk to further aggravate immune pathology. The immune reaction during the early planktonic phase is additionally impaired by the presence of the implant, whose influence has to be considered in immune therapeutic intervention. Furthermore, the type of bacteria plays an important role in the induced immune response; highly virulent strains like *S. aureus* cause strong pro-inflammatory immune reactions, whereas more benign strains such as *S. epidermidis* induce rather moderate and subtle immune responses. All this has to be considered when an immune therapeutic approach is suggested because non-specific boosting of the immune system might end in hyper-inflammation causing tissue damage, while immune inhibition might lead to increased bacterial burden, bacteremia and/or secondary infections. To avoid such conceivable scenarios, we should learn from the lessons already made in sepsis immune-stimulatory therapy which has so far failed to reliably and safely improve patient outcome (139, 140), before introducing immune modulation in the treatment of chronic implant-related bone infections: (1) The immune response is changing throughout the infection, therefore correct timing of therapeutic intervention is indispensable to ensure immune stimulation or inhibition. (2) The immune status of leukocytes can differ depending on the location (lymphoid organs, peripheral blood or site of infection). Systemic immune stimulation/inhibition might not be appropriate and a more tissue/infection site-specific approach should be preferred. Specification can be provided by targeting immune molecules depending on the cell subsets they are preferentially expressed, anatomic prevalence of their expression and/or their distinguished function (141). (3) The immune profile can be highly heterogeneous between patients. Personalized immune therapy should be provided to optimize individual outcome and predictive immune biomarkers should be included in the decision-making for the respective therapeutic target to guarantee responsiveness and minimize adverse effects (135). As the targets of immune modulation have unique functions, combinatory approaches can improve efficacy of immune therapeutic treatment (142). The combination of modulators of innate immune defense with classical ICI targeting adaptive immunity and/or cell-based therapeutic vaccination would allow treatment at multiple levels. However, to ensure optimum patient outcome and safety, immune therapeutics can only be used as medication in addition to current antibiotic and surgical treatment options.

### Targeting Immune Checkpoint Molecules

The first ICI approved for therapy of advanced melanoma was an antibody against CTLA-4 (ipilimumab) in 2011 (143, 144). CTLA-4 is a homologous but antagonistic and competitive receptor for CD28 that has a higher affinity for binding CD80/86 than CD28. Binding of CTL-4 to CD80/86 results in transendocytosis of CD80/86 and inhibition of T cell co-stimulation. Under physiological conditions, CTLA-4 plays an important role in ensuring self-tolerance. The administration of anti-CTLA-4 antibodies proved to be efficient in tumor control but at the same time showed a high incidence of adverse effects and autoimmunity. Activation of this pathway can therefore be a promising approach to treat autoimmune disorders [reviewed in (145)]. Treatment with a soluble CTLA-4-Ig fusion protein (abatacept), which links the extracellular domain of human CTLA-4 to a fragment of the Fc part of human IgG1 (146), was successful in reducing the symptoms of rheumatoid arthritis (RA) (147). However, it aggravated the course of septic arthritis in a mouse model (148). To our knowledge, nothing is known about a potential role of CTLA-4-mediated inhibition of CD28 in chronic implant-related bone infections. Most T cells isolated from blood and tissue of patients undergoing infection-induced revision surgery were shown to be CD28^−^ (92, 93), which might indicate that in chronicity, the majority of effector T cells would not respond to CTLA-4 based therapy. Interestingly, it was shown that binding of CTLA-4 to CD80/86 expressed on the surface of murine bone marrow leukocytes and human blood monocytes directly inhibited RANKL and TNF-mediated differentiation of these cells into osteoclasts *in vitro* and reduced osteoclast formation and bone resorption in an arthritic joint model in mice (149). This suggests that CTLA-4 can be considered as an anti-osteoclastogenic molecule. The inhibitory effect of CD80/86 engagement by CTLA-4 on osteoclastogenesis was further investigated by Bozec et al., who found that induction of apoptosis in osteoclast precursor cells via the IDO/tryptophan pathway was responsible for the reduced osteoclast formation. As expected, the CTLA-4-Ig fusion protein abatacept led to reduced numbers of osteoclast precursor cells and osteoclasts in RA patients and in cell culture experiments. Blocking CTLA-4 with the neutralizing antibody ipilimumab increased the osteoclastogenic potential in humans (150). These data indicate that targeting checkpoint molecules like CTLA-4 provide the opportunity to control osteoclast numbers and bone homeostasis. Still, it has to be considered that in an auto-inflammatory environment, treatment with CTLA-4 is beneficial in reducing osteoclastogenesis, but under infectious conditions it might suppress necessary immune activity and by this potentially aggravate disease progression. Thus, immune checkpoint-mediated inhibition of osteoclastogenesis can be a promising target to decrease inflammation-induced bone resorption and reduce bacterial colonization of damaged tissue, but additional impairment of the immune response has to be excluded.

The best studied immune checkpoint molecule is PD-1 and its ligands PD-L1 and PD-L2, which are targeted to treat T cell dysfunction in cancer and chronic infectious diseases. Compared to CTLA-4, which acts at the level of T cell activation, PD-1 is up-regulated on effector T cells after continuous stimulation. Thus, the PD-1/PD-L1 pathway suppresses activity and function of effector T cells and induces T cell exhaustion (144). Several antibodies targeting the PD-1/PD-L1 pathway have been approved for the treatment of specific cancers and new therapeutics as well as new applications are currently investigated in clinical trials (135). Antibodies against PD-1/PD-L1 have also been transferred into treatment approaches for chronic virus diseases and malaria to improve CD4 and CD8 effector T cell function (136). Additionally, they were shown to have the potential to reverse sepsis-induced immune suppression (137). Negative side effects of PD-1/PD-L1 blockade seem to be less frequent when compared to CTLA-4 treatment. However, nearly half of the patients do not respond to PD-1 blockade alone (136) and combinatory therapy was shown to be more effective, albeit more toxic (151). The role of PD-L2 has not yet been fully clarified: initially, PD-L2 was described as a second ligand for PD-1 that negatively influences T cell immunity (152, 153). A costimulatory function of PD-L2 and the initiation of a Th1 response is also discussed (154). PD-L2 is furthermore suggested to counteract PD-1/PD-L1-mediated T cell exhaustion making a soluble PD-L2 fusion protein an attractive candidate to block the PD-1/PD-L1 pathway (136, 155). In an *in vitro* setting with *S. epidermidis* strains isolated from patients with orthopedic implant loosening, it was shown that after phagocytic uptake SCVs trigger an anti-inflammatory macrophage response with up-regulated PD-L1/L2 expression so that they are able to survive intracellularly without damaging the host cell (156). Whether this also applies to biofilm embedded bacteria has not been investigated yet. Furthermore, MSCs, which are in close contact to the site of bone infections, up-regulate PD-L1/L2 expression and secretion upon stimulation with pro-inflammatory cytokines (157, 158) or induce PD-L1 expression in DCs after exposure to LPS (159). By this, they directly and indirectly inhibit T cell proliferation and function. It can be speculated that the PD-1/PD-L1 pathway might play a role in the persistence of implant-related bone infections. Until now, to our knowledge there is nothing described about an up-regulation of PD-1 on T cells and PD-L1/L2 on host cells associated with biofilm formation during chronic progression of implant-related bone infections. As chronic implant-related bone infections were linked to high numbers of CD28^−^ T cells (92–94) and as it was shown recently that CD28 is indispensable for effectiveness of PD-1 blockade (160, 161), it remains to be seen whether these patients would indeed profit from a PD-1/PD-L1 targeted therapy. OCs were found to mediate their immune suppressive action through galectin-9 and PD-L1 expression and induction of PD-L1 expression on tumor cells in multiple myeloma (162, 163). As OCs are highly present at the site of bone infection, PD-L1 antibodies that can decrease OC-mediated T cell inhibition might enhance T cell immunity in chronic implant-related bone infections.

Lymphocyte activation gene-3 (LAG-3), T cell immunoglobulin and mucin-domain containing protein-3 (TIM-3) and T cell immunoglobulin and ITIM domain (TIGIT) are other immune checkpoint molecules that are currently explored as targets for immune therapy. LAG-3 is up-regulated on CD4 and CD8 T cells as well as on natural killer cells (NK cells). It affects effector T cell function and T_reg_ suppressive activity by binding to MHC class II with higher affinity than CD4 or LSECtin (liver and lymph node sinusoidal endothelial cell C-type lectin). Since LSECtin is involved in antigen uptake (164) and MHC class II is essential for antigen presentation, LAG-3 is suggested to impair the antigen-specific signal in T cell activation [reviewed in (141, 142)]. Indeed, an increased expression of LAG-3 was found on T cells in blood samples of patients suffering from chronic osteomyelitis and was associated with impaired T cell proliferation and function (165). This gives a hint that LAG-3 blockade could be a potential approach for treating chronic implant-associated bone infections. Furthermore, a soluble Lag-3-Ig fusion protein (IMP321) has been shown to lead to APC activation via MHC class II, thus being a candidate to support APC-mediated immunity (166, 167). TIM-3 is expressed on DCs and Mϕs as well as on activated CD4 T cells, predominantly of the Th1 type, CD8 T cells and NK cells [reviewed in (142)]. Via interaction with galectin-9, TIM-3 plays a protective role in autoimmunity by regulating the Th1 response and subsequent macrophage activation (168), triggering cell death (169), and increasing MDSC expansion (170). In cancer and chronic virus infections, high TIM-3 expression was linked to T cell dysfunction. Co-blockade of PD-1 and TIM-3 is superior at improving anti-tumor and anti-viral effector function than PD-1 inhibition alone [reviewed in (141)]. In line with this, TIM-3 expressing T_reg_s showed an increased expression of suppressive molecules and were highly effective in inhibiting Th1/Th17 immune responses (171). Furthermore, high expression levels of TIM-3 on Mϕs are associated either with a quiescent state or an anti-inflammatory (M2) phenotype. Blockade of the TIM-3 pathway therefore may result in a more efficient pro-inflammatory (M1) macrophage response (172) which was shown to reduce biofilm burden in a catheter-associated biofilm infection model in mice (80). Binding of galectin-9 to TIM-3 expressed on osteoclast precursor cells suppressed osteoclast formation and thereby attenuated inflammatory bone loss in adjuvant-induced arthritis (173) indicating a further therapeutic application of the TIM-3/galectin-9 system next to modulating the immune response. TIGIT is a co-inhibitory receptor present on activated T cells, NK cells and T_reg_s and competes with its stimulatory counterpart CD226 for binding of CD155 expressed on APCs, T cells and non-immune cells. CD226 predominantly promotes a Th1/Th17 response with high levels of IFN-γ and IL-17, whereas binding of TIGIT induces a shift toward a Th2 and IL-10 dominated immunity. Therefore, TIGIT interacts with APCs, effector T cells as well as T_reg_s to dampen pro-inflammatory immune responses at multiple levels in favor of a more tolerogenic immune environment [reviewed in (141)]. Prabhakara et al. showed that an early shift from a Th1/Th17 response toward a Th2/T_reg_ immunity was capable of preventing biofilm formation and chronicity of an orthopedic implant infection in mice (98). TIGIT treatment to strengthen a Th2 dominated response might therefore be a supportive strategy in clearing implant-related bone infections at an early stage.

Lag-3, TIM-3, and TIGIT are suggested to regulate immune function at the site of tissue inflammation to inhibit immune pathology, whereas CTLA-4 and PD-1 act more systemically. Because their primary role is to maintain immune homeostasis and self-tolerance in the healthy organism, the first three are predicted to be less toxic (141). Furthermore, due to their specialized roles either at the stage of T cell activation or T cell effector function, molecules from these two groups might exert synergistic effects and provide a more efficient therapeutic outcome when used in combination (142).

### Targeting Innate Immunity

Next to directly improving T cell immunity, another approach is to target innate immune cells and modulate the immune response in a more general way. High MDSC activation and accumulation are found in various cancers where they inhibit T cell proliferation and function, leading to tumor tolerance (174). MDSCs are associated with inflammation-induced tumor progression, as they are activated by pro-inflammatory IL-1β that subsequently induces a tumor-promoting IL-10-dominated environment and an anti-inflammatory (M2) macrophage response (175, 176). Heim et al. showed that MDSC-derived IL-10 is responsible for the anti-inflammatory monocyte response and bacterial persistence in an orthopedic biofilm infection model (87). This indicates that there are some common characteristics between these two chronic diseases. Furthermore, MDSCs express ICI ligands that can directly impair T cell function (177) and also reduce the efficacy of immune checkpoint inhibitors in cancer therapy (178). Therefore, targeting MDSCs in combination with ICI is a promising approach to improve patient outcome (179). Next to the application of approved therapeutics that are effective in reducing MDSC numbers and/or function (e.g., all-trans retinoic acid), the investigation of new drugs that eliminate MDSCs is of high interest. An innate immune checkpoint inhibitor that targets MDSC proliferation and function is currently being investigated in a phase 1 clinical trial (INB_03_) (180). MDSCs contribute to the immune compromised environment in implant-related bone infections and to the chronicity of infection. An early inhibition of MDSC function could be a possible approach to circumvent unwanted immune suppression directly at the onset of infection. In combination with a strict antibiotic treatment this might be able to clear the infection before biofilm manifestation and might prevent bacterial persistence.

Another approach is to directly target macrophage polarization. As tumors and chronic infections are associated with an environment favoring an anti-inflammatory (M2) macrophage response and immune suppression, shifting the balance toward the more pro-inflammatory (M1) macrophage subtype might increase the ability to kill tumor cells and bacteria (181).

Hanke et al. used a cell transfer of exogenously M1-activated Mϕs or administration of a C5a receptor agonist (EP67) in a catheter-associated infection model in mice, which resulted in a pronounced pro-inflammatory Mϕ response and in a reduction of biofilm burden (80). M1 Mϕ not only prevented biofilm formation when injected at an early time point of infection, but were also capable of reducing established biofilms, whereas antibiotic treatment had no effect. This indicates that redirection toward a pro-inflammatory milieu can attenuate mature biofilms. DCs are antigen-presenting cells that activate T cells and induce adaptive defense mechanisms (91). This makes them an attractive tool for immune stimulatory treatment of chronic diseases. Different strategies have been reported and include vaccination strategies with autologous and *ex vivo*-generated DCs that had been stimulated with tumor antigens. After re-injection, these cells can induce an effective anti-cancer immunity through priming of a Th1 and specific cytotoxic T cell response. However, *ex vivo* manipulation is expensive and includes a high risk of infection. The *in vivo* targeting of DCs by antibodies coupled with the respective antigens specifically binding to DC receptors involved in antigen presentation is an attractive alternative [reviewed in (182)]. The role of DCs in the unsuccessful immune response against implant-related bone infections and a potential contribution to biofilm formation has not been investigated so far. Targeting DCs offers the possibility to control the type of T cell response and to induce a biofilm-specific T cell immunity by loading them with biofilm-antigens. Therefore, DC therapy might be an attractive approach to improve a specific host immune defense against implant-related bone infections.

In summary, immune modulation can be a promising approach to restore a desired immune microenvironment during the course of chronic implant-related bone infections: an early immune modulatory intervention might be able to inhibit biofilm formation and inflammation-associated tissue destruction, and might allow the elimination of infection at its onset. After chronic progression of the infection, a comprehensive approach combining surgical removal of infectious tissue, antibiotic treatment and strengthening of the host immune response might improve therapeutic outcome. The combination of rifampin and immune re-activation might be a strategy to eliminate mature biofilms that can increase the chance for surgical regimes with implant retention. After implant exchange, strengthening the specific immunity against the initial infection can help provide an immune response that is able to eliminate potentially remaining bacteria (persister cells) and prevent re-infection. It needs to be clarified in future studies whether the activation of a biofilm-specific immune response by immune therapy is sufficient to combat mature biofilm and other sources of bacterial persistence (SCVs, canalicular propagation) independent of surgical and antibiotic treatment. However, more basic research is needed to address whether an immune modulatory intervention can be a useful treatment strategy in implant-related osteomyelitis. As immune therapy is associated with adverse immune reactions, a safe and beneficial application has to be ensured before applying immune therapeutic approaches into patients with chronic implant-related bone infections.

## Concluding Remarks

Chronic implant-related bone infections are a major problem in orthopedic and trauma surgery. As numbers of joint replacements are rising, complications such as bone infections also increase. Current treatment options are associated with severe consequences for patients and often fail to eliminate the infection. The high risk of chronicity for such infections is due to successful evasion strategies of bacteria with biofilm formation being one major mechanism behind bacterial persistence. The presence of a foreign material facilitates biofilm formation and further supports the persistence of an infection. Thus, there is a high interest to clear infections already at the planktonic stage before biofilm transition occurs and to prevent reinfection after antibiotic and surgical treatment. For this, however, novel therapeutic strategies are required. Immune therapy shows promising results in the treatment of different chronic diseases and strengthening endogenous defense mechanisms could be an attractive new approach for chronic implant-related bone infections. So far, investigations of the immune response against chronic implant-related bone infections demonstrate a discrepancy between a strong pro-inflammatory immune reaction that is associated with osteoclastogenesis and bone destruction, and an immune suppression that potentially impairs successful bacterial killing. Future treatment strategies involving the immune system have to consider this two-sided immune response to avoid adverse reactions. Since the amount of information is limited, the success of immune therapeutic intervention in chronic implant-related bone infections mostly remains speculative and further research is needed to investigate appropriate and safe targets. Furthermore, it has to be clarified if an immune modulatory approach is also capable of targeting bacterial persistence e.g., within biofilms. Immune modulation can serve as an additional and required medical treatment option to restore an effective host response. It is to be hoped that the combination of antibiotic and surgical treatment with immune therapeutic intervention may lead to the successful management of chronic implant-related bone infections in the future.

## Author Contributions

ES reviewed the relevant literature and wrote the manuscript. KK critically revised the manuscript. Both authors have read and approved the manuscript.

### Conflict of Interest Statement

The authors declare that the research was conducted in the absence of any commercial or financial relationships that could be construed as a potential conflict of interest.
